# Inflammatory markers are associated with infertility prevalence: a cross-sectional analysis of the NHANES 2013–2020

**DOI:** 10.1186/s12889-024-17699-4

**Published:** 2024-01-18

**Authors:** Yanfen Chen, Huanying Xu, Jianxing Yan, Qidan Wen, Mingjun Ma, Ningning Xu, Haoxi Zou, Xiaoyan Xing, Yingju Wang, Suzhen Wu

**Affiliations:** 1 Foshan Clinical Medical School of Guangzhou University of Chinese Medicine, Foshan, Guangdong China; 2 First Clinical Medical College of Guangzhou University of Chinese Medicine, Guangzhou, China; 3 TCM Gynecology Department, Foshan Fosun Chancheng Hospital, Chancheng District, Foshan, Guangdong China

**Keywords:** Infertility, National Health and Nutrition Examination Survey (NHANES), Inflammatory markers, Systemic immune inflammation index (SII), Lymphocyte count (LC), Product of platelet and neutrophil count (PPN), Platelet-lymphocyte ratio (PLR), Neutrophil-lymphocyte ratio (NLR), Lymphocyte-monocyte ratio (LMR)

## Abstract

**Background:**

Inflammation exerts a critical role in the pathogenesis of infertility. The relationship between inflammatory parameters from peripheral blood and infertility remains unclear. Aim of this study was to investigate the association between inflammatory markers and infertility among women of reproductive age in the United States.

**Methods:**

Women aged 20–45 were included from the National Health and Nutrition Examination Survey (NHANES) 2013–2020 for the present cross-sectional study. Data of reproductive status was collected from the Reproductive Health Questionnaire. Six inflammatory markers, systemic immune inflammation index (SII), lymphocyte count (LC), product of platelet and neutrophil count (PPN), platelet-lymphocyte ratio (PLR), neutrophil–lymphocyte ratio (NLR) and lymphocyte-monocyte ratio (LMR) were calculated from complete blood counts in mobile examination center. Survey-weighted multivariable logistic regression was employed to assess the association between inflammatory markers and infertility in four different models, then restricted cubic spline (RCS) plot was used to explore non-linearity association between inflammatory markers and infertility. Subgroup analyses were performed to further clarify effects of other covariates on association between inflammatory markers and infertility.

**Results:**

A total of 3,105 women aged 20–45 was included in the final analysis, with 431 (13.88%) self-reported infertility. A negative association was found between log2-SII, log2-PLR and infertility, with an OR of 0.95 (95% CI: 0.78,1.15; *p* = 0.60), 0.80 (95% CI:0.60,1.05; *p* = 0.10), respectively. The results were similar in model 1, model 2, and model 3. Compared with the lowest quartile (Q1), the third quartile (Q3) of log2-SII was negatively correlation with infertility, with an OR (95% CI) of 0.56 (95% CI: 0.37,0.85; *p* = 0.01) in model 3. Similarly, the third quartile (Q3) of log2-PLR was negatively correlation with infertility, with an OR (95% CI) of 0.61 (95% CI: 0.43,0.88; *p* = 0.01) in model 3. No significant association was observed between log2-LC, log2-PPN, log2-NLR, log2-LMR and infertility in model 3. A similar U-shaped relationship between log2-SII and infertility was found (*p* for non-linear < 0.05). The results of subgroup analyses revealed that associations between the third quartile (Q3) of log2-SII, log2-PLR and infertility were nearly consistent.

**Conclusion:**

The findings showed that SII and PLR were negatively associated with infertility. Further studies are needed to explore their association better and the underlying mechanisms.

**Supplementary Information:**

The online version contains supplementary material available at 10.1186/s12889-024-17699-4.

## Introduction

Global disease burden of infertility has been increasing, as rate of female infertility increased by 14.962% throughout the period from 1990 to 2017 [1]. Among women diagnosed with infertility, up to 30% are considered unexplained infertility following standard investigation, which then would be advised to undergo cost prohibitive assisted reproductive technology with risks of adverse pregnancy and childbirth outcomes or laparoscopy combined with hysteroscopy based on age, ovarian reserve, infertility duration and other factors of women [2, 3]. Infertility should be regarded as more than reproductive health problem considering its public health economic burden and harmful influences on women’s psychological distress, as well as marital discord. Significant evidence has suggested that systemic or local inflammation and immune response are considered one of the most critical factors contributing to unexplained infertility [4]. Immune cells (including macrophages, natural killer cells, dendritic cells, and T cells) and immune regulatory molecules (such as IL-6, IL-10, TNF-α, and TGF-β1) maintaining homeostasis of endocrine display abnormal activity in women with diseases related to infertility [5, 6].

Systemic immune inflammation index (SII), lymphocyte count (LC), product of platelet and neutrophil count (PPN), platelet-lymphocyte ratio (PLR), neutrophil–lymphocyte ratio (NLR), and lymphocyte-monocyte ratio (LMR) calculated from peripheral blood cell counts are emerging inflammatory markers for diseases status or prognosis prediction. There is growing interest in research aimed at identifying value of complete blood cell counts derived inflammatory indicators because of their low cost-effective, rapid, and convenient process of blood draw and test. SII has been developed as an integrated and novel inflammatory indicator, while recent study confirmed the close relationship between SII and gynecological and breast cancers [7]. Earlier study demonstrated that the number of lymphocytes, the portion of CD4 + T lymphocytes, and NK cells were significantly elevated in women diagnosed with Polycystic Ovary Syndrome (PCOS) and that CD4 + T cells and NK cells were independent risk factors for PCOS [8], indicating peripheral blood inflammatory-immune cells as promising predictor of infertility among these patients. Aberrant function and frequency of lymphocytes have been accepted as partial pathogenesis of infertility and disturbed pregnancy [9], thus lymphocyte immunotherapy could be utilized to enhance maternal immune system against the paternal antigens with its promising effects on dysfunction of aberrant change of peripheral lymphocyte subsets. PPN is calculated as peripheral platelet count × neutrophil count. It has been reported that PPN was associated with female estradiol and bone mineral density. Researchers also found that SII, NLR was positively associated with female estradiol [10, 11].

Previous study demonstrated that NLR level increased in patients with PCOS and was positively correlated with insulin resistance. Furthermore, researchers considered that NLR might be a more sensitive marker to present low grade inflammation in patients with PCOS [12]. LMR has been introduced as an effective indicator for prognosis of patients with various carcinoma, acute ischemic stroke, and hypertension by recent studies [13–18]. PLR is ratio index calculated by inflammatory activators platelets and inflammatory regulators lymphocytes, which was positively associated with asthma prevalence [19] and adverse outcomes in acute exacerbations of chronic obstructive pulmonary disease [20].

Dysfunction and changed frequency of immune cells are closely related to infertility. However, association between SII, LC, PPN, PLR, NLR, LMR and infertility have not been reported yet. Therefore, this study aims to investigate association between inflammatory parameters and female infertility based on the National Health and Nutrition Examination Survey (NHANES) 2013–2020 data, hoping to provide new insights in inflammatory markers for female infertility.

## Methods

### Data source and population selection

NHANES, a continuous health and nutritional status program of adults and children in the United States, has been conducted as surveys including demographic, socioeconomic, dietary and health questions. NHANES was approved by the National Center for Health Statistics Institutional Review Board (https://www.cdc.gov/nchs/nhanes/irba98.htm), and informed consent of all individuals were obtained before participation in this program.

**Fig. 1:**
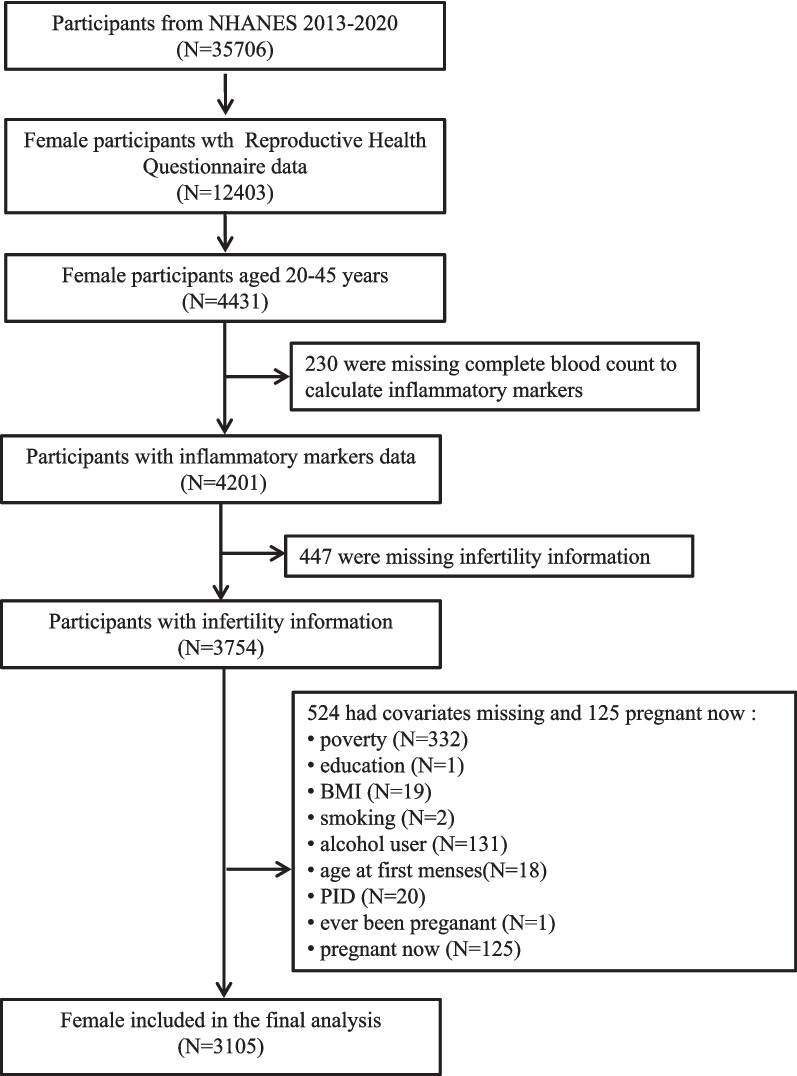
Flowchart of participants selection. Legend: Abbreviations: NHANES, National Health and Nutrition Examination Survey

A total of 4,431 female participants aged 20–45 with finished Reproductive Health Questionnaire data was chosen from 35,706 individuals in the NHANES 2013–2020. Then, those with missing data to calculate inflammatory markers (*n* = 230), infertility information (*n* = 447), and other variables (*n* = 649) were excluded. Finally, 3,105 female participants were included in our analyses (Fig. 1).

### Definition of infertility and inflammatory markers

Self-reported infertility was determined according to answer to the specific two questions (question RHQ074 and question RHQ076) from the Reproductive Health Questionnaire. Question RHQ074: “Have you ever attempted to become pregnant over a period of at least a year without becoming pregnant?” Question RHQ076: “Have you ever been to a doctor or other medical provider because you have been unable to become pregnant?” Any woman who self-reported “Yes” to either of these questions was considered to have a history of infertility. Women who answered “Yes” were divided into the “infertility group”, while those who answered “No” were to the “non-infertility group”.

We collected lymphocyte count (LC), platelet count (PC), neutrophil count (NC) and monocyte count (MC) expressed in × 1000 cells/μ l from complete blood count to calculate the following inflammatory markers: SII was calculated as PC * NC/LC, PPN was the product of platelet and neutrophil, PLR was platelet to lymphocyte ratio, NLR was neutrophil to lymphocyte ratio, and LMR was lymphocyte to monocyte ratio.

### Covariates

According to clinical practice, previous literature [21–26], and available in the NHANES database, we selected age, body mass index (BMI), race, marital status, education level, smoking status, alcohol user, income to poverty ratio (PIR), pelvic infection disease (PID), and age of menarche as covariates, aimed to control potential confounding bias in this study. Age was divided into two groups based on clinical significance (< 35, and ≥ 35 years). BMI was categorized into normal (< 25 kg/m^2^), overweight (25–30 kg/m^2^), and obesity (≥ 30 kg/m^2^) by clinical significance. Race was classified as “Mexican”, “Hispanic”, “White”, “Black”, and “Other race”. Marital status was classified as “Married/Living with partner”, and “Living alone”. Education level was divided into three groups as “Some college or AA degree above”, “High school/GED”, and “Less than 11th grade”. Smoking status was defined as “Yes” or “No” based on self-reported having smoked at least 100 cigarettes in their lifetime. The classification of alcohol user was determined through self-reporting as follows: heavy, self-reported ≥ 4 drinks every day; mild, self-reported ≤ 3 drinks every day; former, did not drink last year but drank ≥ 12 drinks in a lifetime or self-reported ≥ 12 drinks in 1 year and did not drink last year; never, self-reported < 12 drinks in a lifetime. The family PIR was categorized into three degrees (< 1.5, 1.5–3.5, and ≥ 3.5). PID was determined by the self-reported questions from Reproductive Health Questionnaire (RHQ078): Ever been treated for a pelvic infection/PID? The age of menarche was classified into two levels (< 15, and ≥ 15 years) by clinical practice.

### Statistical analysis

Weight used for analysis was chosen based on instructions on the NHANES database. Weighting (2022), available at: https://wwwn.cdc.gov/nchs/nhanes/tutorials/Weighting.aspx, suggested that mobile examination center exam weight (WTMEC2YR) should be applied as complete blood count measured in the mobile examination center. Categorical variables were expressed as frequency (weighted proportion, %), and continuous variables were expressed as weighted mean (standard deviation, SD). Chi-square test (categorical variables) or Wilcoxon test (continuous variables) was used to compare significant differences between infertility and non-infertility groups.

As SII, LC, PPN, PLR, NLR and LMR from individuals included in the present study were right-skewed distribution (shown in Supplementary Fig. 1), SII, LC, PPN, PLR, NLR, and LMR were log2-transformed before data analysis (shown in Supplementary Fig. 2). Survey-weighted multivariable logistic regression was used to assess the association between inflammatory markers and infertility in four different models. No covariate was adjusted in crude model and age(continuous) was adjusted in model 1. Model 2 was adjusted for variables including age(continuous), BMI (continuous), race (Mexican, Hispanic, White, Black, and Other race), marital status (living alone, and married/living with partner), education (Some college or AA degree above, High school/GED, and Less than 11th grade), smoking status (No, Yes), alcohol user (never, former, mild, and heavy) and PIR (< 1.5, 1.5–3.5, and ≥ 3.5). Then based on model 2, we adjusted PID (No,Yes) and age of menarche (< 15 years or ≥ 15 years) in model 3. We then performed restricted cubic spline (RCS) plot to assess the potential non-linearity association between infertility and inflammatory markers. Furthermore, we performed subgroup and interaction analyses by all covariates to ensure the robustness of the result. R (version 4.3.1, http://www.R-project.org) was utilized to analyze data obtained from NHANES datasets, two-tailed *P* < 0.05 was of statistical significance.

## Results

### Baseline characteristics

A total sample of 3105 women represented a population of 40,670,393 women in the United States, with 431 (13.88%) self-reported infertility. Baseline characteristics of included women from NHANES 2013 to 2020 were shown in Table 1. The mean age of non-infertility and infertility women was 32.08 ± 0.22 and 35.18 ± 0.47 years, and the mean BMI was 29.19 ± 0.24 and 31.89 ± 0.63 kg/m^2^, respectively. Compared with non-infertility women, infertility women were more likely to be older, overweight, married or living with a partner, smoking, and history of pelvic infection disease (*p* < 0.05). No significant differences were found between the two groups in terms of race, education, alcohol user, PIR, and age of  menarche. The mean log2-LC of infertility women was 1.22 (0.03) × 1000 cells/μl, significantly higher than those of non-infertility women. While no significant differences were found in log2-SII, log2-PPN, log2-PLR, log2-NLR, and log2-LMR between infertility women and non-infertility women.

**Table 1:** Baseline characteristics of included women from NHANES 2013 to 2020

Variables	Total (*n* = 3105)	Non-infertility (*n* = 2674)	Infertility (*n* = 431)	*P*
**Age, years, (%)**				< 0.01
< 35	1739 (57.51)	1552 (59.74)	187 (44.23)	
≥ 35	1366 (42.49)	1122 (40.26)	244 (55.77)	
**BMI, kg/m**^**2**^**, n (%)**				< 0.01
< 25	1032 (35.84)	916 (37.28)	116 (27.28)	
25–30	731 (23.66)	652 (24.42)	79 (19.15)	
≥ 30	1342 (40.49)	1106 (38.29)	236 (53.56)	
**Race, n (%)**				0.33
White	1060 (58.82)	900 (58.21)	160 (62.45)	
Black	709 (12.60)	613 (12.69)	96 (12.07)	
Mexican	496 (11.25)	428 (11.25)	68 (11.23)	
Hispanic	310 (7.34)	276 (7.65)	34 (5.52)	
Other race	530 (9.98)	457 (10.19)	73 (8.73)	
**Marital status, n (%)**				< 0.01
Married/Living with partner	1753 (59.81)	1446 (57.04)	307 (76.30)	
Living alone	1352 (40.19)	1228 (42.96)	124 (23.70)	
**Education, n (%)**				0.79
Less than 11th grade	432 (9.51)	374 (9.66)	58 (8.65)	
High school or GED	580 (18.38)	499 (18.26)	81 (19.05)	
Some college or AA degree above	2093 (72.11)	1801 (72.08)	292 (72.30)	
**Smoking status, n (%)**				0.02
No	2186 (67.77)	1907 (68.78)	279 (61.79)	
Yes	919 (32.23)	767 (31.22)	152 (38.21)	
**Alcohol user, n (%)**				0.15
Never	466 (11.28)	417 (11.73)	49 (8.67)	
Former	150 (4.03)	125 (3.72)	25 (5.88)	
Mild	1687 (56.54)	1450 (56.75)	237 (55.30)	
Heavy	802 (28.15)	682 (27.81)	120 (30.15)	
**Poverty to income ratio, n (%)**				0.08
< 1.5	1243 (30.18)	1097 (31.12)	146 (24.65)	
1.5–3.5	1000 (32.31)	854 (31.85)	146 (35.02)	
≥ 3.5	862 (37.51)	723 (37.03)	139 (40.33)	
**Pelvic Infection Disease, n (%)**				< 0.01
No	2945 (95.68)	2553 (96.43)	392 (91.18)	
Yes	160 (4.32)	121 (3.57)	39 (8.82)	
**Age of menarche, years, n (%)**				0.65
< 15	2541 (82.36)	2190 (82.22)	351 (83.20)	
≥ 15	564 (17.64)	484 (17.78)	80 (16.80)	
**Inflammatory index**^**a**^				
log2-SII, mean (SD)	8.94 (0.02)	8.95 (0.02)	8.92 (0.04)	0.60
log2-LC, 1000 cells/μl, mean (SD)	1.16 (0.01)	1.16 (0.01)	1.22 (0.03)	0.05
log2-PPN, mean (SD)	10.90 (0.02)	10.89 (0.02)	10.95 (0.04)	0.15
log2-PLR, mean (SD)	6.85 (0.01)	6.85 (0.01)	6.81 (0.02)	0.10
log2-NLR, mean (SD)	1.72 (0.01)	1.73 (0.01)	1.71 (0.02)	0.47
log2-LMR, mean (SD)	2.08 (0.01)	2.08 (0.01)	2.09 (0.03)	0.77

Continuous variables were expressed as weighted mean (standard deviation, SD), categorical variables were expressed as number (weighted proportion, %)

*Abbreviations*: *BMI* body mass index, *SII* systemic immune inflammation index, *LC* lymphocyte count, *PPN* product of platelet and neutrophil count, *PLR* platelet-lymphocyte ratio, *NLR* neutrophil–lymphocyte ratio, *LMR* lymphocyte-monocyte ratio

^a^Given the skewed distribution of these inflammatory indicators, SII, LC, PPN, PLR, NLR and LMR were log2-transformed before data analysis

### Associations between inflammatory markers and infertility

The associations between inflammatory markers and infertility were shown in Table 2. In crude model, log2-LC was positively associated with infertility (OR 1.42; 95% CI: 1.00, 2.00; *p* = 0.05), and the results were stable in model 1, with OR of 1.51 (95% CI: 1.06, 2.16; *p* = 0.02). However, the results were not significantly stable in model 2 and model 3, with OR of 1.33 (95% CI: 0.93, 1.90; *p* = 0.12), 1.33 (95% CI: 0.93, 1.92; *p* = 0.12). No significant association was observed between log2-SII, log2-PPN, log2-PLR, log2-NLR, log2-LMR and infertility in crude model, with OR of 0.95 (95% CI: 0.78, 1.15; *p* = 0.60), 1.17 (95% CI: 0.94, 1.47; *p* = 0.15), 0.80 (95% CI: 0.60, 1.05; *p* = 0.10), 0.87 (95% CI: 0.58, 1.29; *p* = 0.48), and 1.05 (95% CI: 0.78, 1.41; *p* = 0.77), respectively. The results were similar in model 1, model 2, and model 3.

**Table 2:** Associations between inflammatory markers and infertility

Inflammatory markers	Total, n	Infertility, n	Crude Model		Model 1		Model 2		Model 3	
			**OR (95%CI)**	***P***	**OR (95%CI)**	***P***	**OR (95%CI)**	***P***	**OR (95%CI)**	***P***
**log2-SII**	3105	431	0.95 (0.78, 1.15)	0.60	0.91 (0.74, 1.10)	0.32	0.82 (0.66, 1.02)	0.07	0.82 (0.66, 1.02)	0.08
Q1 [5.40, 8.45]	778	117	ref		ref		ref		ref	
Q2 (8.45, 8.93]	774	106	0.87 (0.61, 1.25)	0.45	0.81 (0.57, 1.17)	0.26	0.77 (0.53, 1.11)	0.16	0.77 (0.52, 1.12)	0.17
Q3 (8.93, 9.38]	776	92	0.71 (0.49, 1.03)	0.07	0.65 (0.44, 0.95)	***0.03***	0.57 (0.38, 0.85)	***0.01***	0.56 (0.37, 0.85)	***0.01***
Q4 (9.38, 12.51]	777	116	1.08 (0.76, 1.53)	0.65	0.97 (0.68, 1.38)	0.87	0.83 (0.55, 1.25)	0.37	0.83 (0.55, 1.27)	0.38
**log2-LC**	3105	431	1.42 (1.00, 2.00)	***0.05***	1.51 (1.06, 2.16)	***0.02***	1.33 (0.93, 1.90)	0.12	1.33 (0.93, 1.92)	0.12
Q1 [-1.00, 0.93]	959	124	Ref		Ref		Ref		Ref	
Q2 (0.93, 1.20]	768	86	0.77 (0.53, 1.11)	0.16	0.79 (0.55, 1.12)	0.17	0.72 (0.51, 1.03)	0.07	0.72 (0.51, 1.03)	0.07
Q3 (1.20, 1.43]	603	93	1.06 (0.75, 1.50)	0.74	1.08 (0.75, 1.57)	0.67	0.97 (0.67, 1.40)	0.86	0.98 (0.68, 1.40)	0.90
Q4 (1.43, 2.72]	775	128	1.46 (1.04, 2.03)	***0.03***	1.55 (1.11, 2.18)	***0.01***	1.36 (0.95, 1.94)	0.09	1.35 (0.94, 1.94)	0.10
**log2-PPN**	3105	431	1.17 (0.94, 1.47)	0.15	1.17 (0.92, 1.47)	0.19	1.01 (0.79, 1.28)	0.95	1.01 (0.79, 1.28)	0.94
Q1 [8.00, 10.47]	778	95	Ref		Ref		Ref		Ref	
Q2 (10.47, 10.89]	774	121	1.52 (1.02, 2.26)	0.04	1.57 (1.05, 2.35)	***0.03***	1.38 (0.92, 2.08)	0.11	1.38 (0.92, 2.07)	0.11
Q3 (10.89, 11.30]	777	101	1.13 (0.77, 1.66)	0.53	1.13 (0.77, 1.66)	0.52	0.96 (0.64, 1.45)	0.85	0.96 (0.63, 1.44)	0.82
Q4 (11.30, 13.42]	776	114	1.50 (1.04, 2.15)	***0.03***	1.52 (1.03, 2.24)	***0.03***	1.19 (0.79, 1.79)	0.39	1.20 (0.80, 1.79)	0.37
**log2-PLR**	3105	431	0.80 (0.60, 1.05)	0.10	0.74 (0.56, 0.98)	***0.04***	0.76 (0.57, 1.01)	0.06	0.76 (0.57, 1.00)	***0.05***
Q1 [3.68, 6.56]	779	123	Ref		Ref		Ref		Ref	
Q2 (6.56, 6.85]	773	116	0.82 (0.58, 1.14)	0.23	0.82 (0.58, 1.17)	0.26	0.82 (0.57, 1.17)	0.26	0.83 (0.57, 1.19)	0.30
Q3 (6.85, 7.16]	777	92	0.64 (0.45, 0.89)	***0.01***	0.61 (0.43, 0.87)	***0.01***	0.60 (0.42, 0.87)	***0.01***	0.61 (0.43, 0.88)	***0.01***
Q4 (7.16, 8.82]	776	100	0.82 (0.57, 1.17)	0.26	0.75 (0.52, 1.07)	0.11	0.76 (0.52, 1.11)	0.15	0.75 (0.51, 1.10)	0.13
**log2-NLR**	3105	431	0.87 (0.58, 1.29)	0.48	0.79 (0.53, 1.19)	0.26	0.73 (0.46, 1.16)	0.18	0.73 (0.46, 1.18)	0.19
Q1 [0.61, 1.45]	778	115	Ref		Ref		Ref		Ref	
Q2 (1.45, 1.69]	782	104	0.84 (0.60, 1.18)	0.30	0.78 (0.55, 1.10)	0.15	0.72 (0.51, 1.02)	0.06	0.71 (0.50, 1.02)	0.06
Q3 (1.69, 1.93]	772	104	0.86 (0.59, 1.25)	0.43	0.76 (0.53, 1.11)	0.15	0.72 (0.49, 1.06)	0.10	0.72 (0.49, 1.06)	0.10
Q4 (1.93, 3.86]	773	108	0.98 (0.70, 1.36)	0.89	0.89 (0.64, 1.23)	0.46	0.81 (0.57, 1.16)	0.24	0.81 (0.56, 1.16)	0.24
**log2-LMR**	3105	431	1.05 (0.78, 1.41)	0.77	1.07 (0.79, 1.45)	0.64	0.99 (0.72, 1.37)	0.97	1.01 (0.73, 1.39)	0.96
Q1 [0.11, 1.81]	770	96	Ref		Ref		Ref		Ref	
Q2 (1.81, 2.12]	767	114	1.53 (1.01, 2.30)	***0.04***	1.48 (0.99, 2.22)	0.05	1.49 (0.99, 2.23)	0.06	1.47 (0.98, 2.20)	0.06
Q3 (2.12, 2.43]	827	119	1.33 (0.87, 2.03)	0.19	1.28 (0.83, 1.98)	0.26	1.18 (0.74, 1.89)	0.47	1.20 (0.75, 1.91)	0.44
Q4 (2.43, 4.03]	741	102	1.14 (0.78, 1.65)	0.49	1.18 (0.81, 1.71)	0.39	1.12 (0.76, 1.63)	0.56	1.12 (0.77, 1.64)	0.54

Crude model was not adjusted. Model 1 was adjusted age(continuous). Model 2 was adjusted for model 1 + BMI (continuous), race (Mexican, Hispanic, White, Black, and Other race), marital status (living alone, and married/living with partner), education (Some college or AA degree above, High school or GED, and Less than 11th grade), smoking status (No, Yes), alcohol user (never, former, mild, and heavy) and PIR (< 1.5, 1.5–3.5, and ≥ 3.5). Model 3 was adjusted for model 2 + PID (No, Yes), and age of menarche (< 15 years or ≥ 15 years). *P* value in bold indicates statistical significance

*Abbreviation*: *OR* odds ratios, *CI* confidence interval, *ref* reference group, *SII* systemic immune inflammation index, *LC* lymphocyte count, *PPN* product of platelet and neutrophil count, *PLR* platelet-lymphocyte ratio, *NLR* neutrophil–lymphocyte ratio, *LMR* lymphocyte-monocyte ratio, *Q1* the first quartile, *Q2* the second quartile, *Q3* the third quartile, *Q4* the highest quartile

Furthermore, when these inflammatory markers were transformed from continuous variables to categorical variables by quartile, we found that women in the highest log2-LC quarter group (Q4) showed significantly highest risk of infertility compared with the lowest log2-LC quarter group (Q1) in both crude model (OR 1.46; 95% CI: 1.04, 2.03; *p* = 0.03) and model 1 (OR 1.55; 95% CI: 1.11, 2.18; *p* = 0.01). However, the highest quartile (Q4) of log2-LC were not significantly positively correlate with infertility in model 2 and model 3, with OR of 1.36 (95% CI:  0.95, 1.94; *p* = 0.09), 1.35 (95% CI: 0.94, 1.94; *p* = 0.10). The results of the association between log2-PPN by quartile and infertility were similar with log2-LC. Compared with the lowest quartile (Q1), the third quartile (Q3) of log2-SII was negatively correlation with infertility, with an OR (95% CI) of 0.71 (95% CI: 0.49, 1.03; *p* = 0.07) in crude model, 0.65 (95% CI: 0.44, 0.95; *p* = 0.03) in model 1, 0.57 (95% CI:  0.38, 0.85; *p* = 0.01) in model 2, and 0.56 (95% CI: 0.37, 0.85; *p* = 0.01) in model 3. While the ORs (95% CIs) for infertility with log2-SII levels in Q2 and Q4 in model 3 were 0.77 (95% CI: 0.52, 1.12; *p* = 0.17), and 0.83 (95% CI: 0.55, 1.27; *p* = 0.38), respectively. Similarly, the results of the association between log2-PLR by quartile and infertility were similar with log2-SII. No significant association was observed between log2-NLR, log2-LMR by quartile and infertility in model 3.

**Fig. 2:**
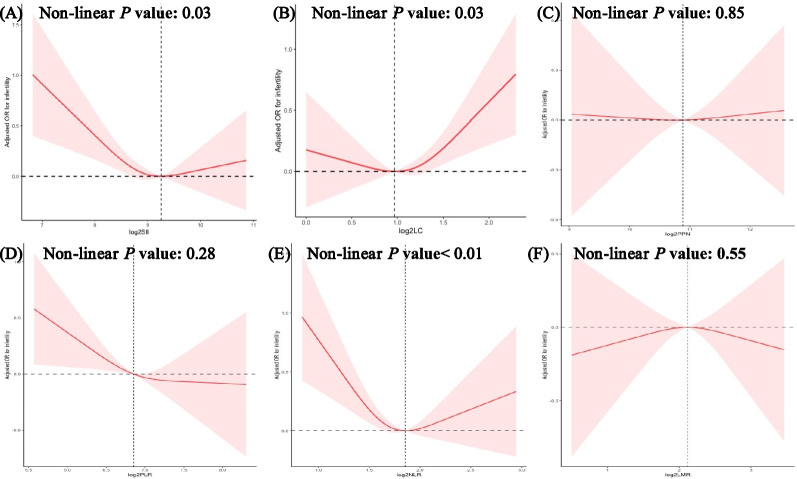
Restricted cubic spline plot of the association between inflammatory markers and infertility. Legend: **A** log2-transformed SII; **B** log2-transformed LC; **C** log2-transformed PPN; **D** log2-transformed PLR; **E** log2-transformed NLR; **F** log2-transformed LMR. Abbreviation: SII, systemic immune inflammation index; LC, lymphocyte count; PPN, product of platelet and neutrophil count; PLR, platelet to lymphocyte ratio; NLR, neutrophil to lymphocyte ratio; LMR, lymphocyte to monocyte ratio

Restricted cubic spline (RCS) plot was performed to assess the potential non-linearity of the association between infertility and inflammatory markers. In Fig. 2, a similar U-shaped relationship between log2-SII and infertility was found (*p* for non-linear < 0.05). A non-linear relationship between log2-LC, log2-NLR and infertility was found (*p* for non-linear < 0.05), however, there were no significant association between log2-LC, log2-NLR by quartile and infertility in model 3 shown in Table 2. Of note, linear relationship could be observed between log2-PPN, log2-PLR, log2-LMR and infertility (*p* > 0.05).

### Subgroup analysis 

We performed subgroup analyses to assess the stability of the association between log2-SII, log2-PLR by quartile and the risk of infertility in different populations based on age, BMI, race, marital status, education, smoking status, alcohol user, PIR, PID, and age of menarche (Tables 3 and 4). All covariates in each subgroup analysis model were adjusted, except the stratification variable itself. Compared with the lowest quartile (Q1), the third quartile (Q3) of log2-SII was associated with a decreased risk of infertility among those with BMI < 25 kg/m^2^ (OR, 0.43; 95% CI: 0.24, 0.79), Black (OR, 0.46; 95% CI: 0.24, 0.90), living alone (OR, 0.38; 95% CI: 0.21, 0.67), Some college or AA degree above (OR, 0.66; 95% CI: 0.46, 0.94), no history of PID (OR, 0.69; 95% CI: 0.50, 0.93). Significant interactions were observed in BMI, race, marital status, education, alcohol user, PIR, PID, and age of menarche (all *p* for interaction < 0.05), indicating that the negative correlation between log2-SII and infertility was also affected by the interaction among those different subgroups. Compared with the lowest quartile (Q1), the third quartile (Q3) of log2-PLR was consistently associated with a decreased risk of infertility among those aged < 35 years (OR, 0.59; 95% CI: 0.38, 0.91), BMI ≥ 30.0 kg/m^2^ (OR, 0.57; 95% CI: 0.38, 0.86), White (OR, 0.63; 95% CI: 0.40, 1.01), living alone (OR, 0.58; 95% CI: 0.34, 0.97), Some college or AA degree above (OR, 0.66; 95% CI: 0.46, 0.94), smoking(OR, 0.60; 95% CI: 0.36, 0.99), PIR among 1.5–3.5 (OR, 0.53; 95% CI: 0.32–0.88), no history of PID (OR, 0.71; 95% CI: 0.52, 0.96), and age of menarche < 15 years (OR, 0.70; 95% CI: 0.50, 0.97). Significant interactions were observed in BMI, race, education, PIR, and age of menarche (all *p* for interaction < 0.05), indicating that the negative correlation between log2-PLR and infertility was also affected by the interaction among those different subgroups. Furthermore, the association between log2-LC, log2-PPN, log2-NLR, log2-LMR by quartile and the risk of infertility in different subgroups were not significant (shown in Supplementary Tables 1, 2, 3 and 4).

**Table 3:** Subgroup analyses for the relationship between log2-SII and infertility

Subgroups	Q1 [5.40,8.45]	Q2 (8.45,8.93]	Q3 (8.93,9.38]	Q4 (9.38,12.51]	*P* for interaction
**Age, years**					0.24
< 35	ref	0.77 (0.51, 1.18)	0.67 (0.44, 1.03)	0.99 (0.66, 1.48)	
≥ 35	ref	0.94 (0.64, 1.40)	0.80 (0.53, 1.21)	0.90 (0.61, 1.32)	
**BMI, kg/m**^**2**^					** < *0.01***
< 25	ref	0.78 (0.47, 1.28)	0.43 (0.24, 0.79)	0.94 (0.56, 1.59)	
25–30	ref	0.55 (0.27, 1.11)	1.03 (0.55, 1.92)	1.04 (0.56, 1.94)	
≥ 30	ref	1.04 (0.69, 1.58)	0.77 (0.50, 1.18)	0.86 (0.57, 1.29)	
**Race**					** < *0.01***
White	ref	0.97 (0.60, 1.57)	0.70 (0.42, 1.16)	0.98 (0.61, 1.59)	
Black	ref	0.60 (0.34, 1.06)	0.46 (0.24, 0.90)	0.55 (0.29, 1.03)	
Mexican	ref	2.57 (1.05, 6.28)	1.52 (0.59, 3.93)	2.81 (1.16, 6.80)	
Hispanic	ref	1.58 (0.55, 4.54)	1.09 (0.37, 3.23)	0.91 (0.30, 2.75)	
Other race	ref	0.46 (0.20, 1.07)	1.09 (0.56, 2.13)	1.21 (0.62, 2.37)	
**Marital status**					** < *0.01***
Married/Living with partner	ref	0.98 (0.68, 1.39)	1.02 (0.71, 1.45)	1.20 (0.85, 1.69)	
Living alone	ref	0.74 (0.46, 1.21)	0.38 (0.21, 0.67)	0.67 (0.41, 1.09)	
**Education**					***0.01***
Less than 11th grade	ref	1.19 (0.54, 2.63)	1.68 (0.77, 3.66)	1.01 (0.45, 2.29)	
High school or GED	ref	1.05 (0.55, 2.01)	0.70 (0.35, 1.40)	0.84 (0.44, 1.61)	
Some college or AA degree above	ref	0.81 (0.58, 1.15)	0.66 (0.46, 0.94)	1.04 (0.74, 1.45)	
**Smoking status**					0.11
No	ref	0.82 (0.58, 1.16)	0.70 (0.49, 1.01)	1.03 (0.74, 1.45)	
Yes	ref	1.08 (0.66, 1.76)	0.89 (0.53, 1.47)	0.89 (0.55, 1.45)	
**Alcohol user**					***0.01***
Never	ref	0.68 (0.29, 1.57)	1.14 (0.52, 2.49)	0.81 (0.35, 1.88)	
Former	ref	1.25 (0.36, 4.30)	1.03 (0.30, 3.51)	1.46 (0.44, 4.83)	
Mild	ref	0.99 (0.67, 1.44)	0.73 (0.48, 1.09)	1.13 (0.78, 1.64)	
Heavy	ref	0.76 (0.44, 1.31)	0.60 (0.35, 1.04)	0.72 (0.42, 1.22)	
**Poverty to income ratio**					***0.01***
< 1.5	ref	1.28 (0.80, 2.04)	0.86 (0.51, 1.43)	1.02 (0.62, 1.66)	
1.5–3.5	ref	0.79 (0.48, 1.28)	0.71 (0.43, 1.16)	0.78 (0.48, 1.27)	
≥ 3.5	ref	0.65 (0.38, 1.12)	0.69 (0.41, 1.15)	1.18 (0.73, 1.92)	
**Pelvic Infection Disease**					***0.03***
No	ref	0.87 (0.65, 1.17)	0.69 (0.50, 0.93)	0.95 (0.71, 1.26)	
Yes	ref	1.24 (0.40, 3.90)	2.24 (0.75, 6.73)	1.83 (0.60, 5.62)	
**Age of menarche, years**					***0.02***
< 15	ref	1.04 (0.76, 1.42)	0.82 (0.59, 1.14)	1.13 (0.83, 1.54)	
≥ 15	ref	0.47 (0.24, 0.93)	0.57 (0.30, 1.07)	0.58 (0.30, 1.11)	

The model was adjusted for age (categorical), BMI (categorical), race (Mexican, Hispanic, White, Black, and Other race), marital status (living alone, and married/living with partner), education (Some college or AA degree above, High school or GED, and Less than 11th grade), smoking status (No, Yes), alcohol user (never, former, mild, and heavy) and PIR (< 1.5, 1.5–3.5, and ≥ 3.5), PID (No, Yes), and age of menarche (< 15 years or ≥ 15 years). All covariates in the subgroup analysis models were adjusted, excepting the stratification variable itself (for example, “age” was not included as a covariate in the age subgroup). *P* value in bold indicates statistical significance

*Abbreviation*: *BMI* body mass index, *OR* odds ratios, *CI* confidence interval, *ref* reference group, *SII* systemic immune inflammation index, *Q1* the first quartile, *Q2* the second quartile, *Q3* the third quartile, *Q4* the highest quartile

**Table 4:** Subgroup analyses for the relationship between log2-PLR and infertility

Subgroups	Q1 [3.68, 6.56]	Q2 (6.56, 6.85]	Q3 (6.85, 7.16]	Q4 (7.16, 8.82]	*P* for interaction
**Age, years**					0.06
< 35	ref	1.02 (0.69, 1.49)	0.59 (0.38, 0.91)	0.62 (0.40, 0.98)	
≥ 35	ref	0.85 (0.57, 1.26)	0.79 (0.53, 1.18)	0.81 (0.56, 1.19)	
**BMI, kg/m**^**2**^					***0.03***
< 25	ref	0.83 (0.48, 1.43)	0.84 (0.49, 1.43)	0.79 (0.46, 1.35)	
25–30	ref	1.24 (0.65, 2.38)	1.05 (0.53, 2.07)	1.24 (0.64, 2.41)	
≥ 30	ref	0.91 (0.63, 1.32)	0.57 (0.38, 0.86)	0.68 (0.46, 1.01)	
**Race**					** < *0.01***
White	ref	0.64 (0.40, 1.02)	0.63 (0.40, 1.01)	0.79 (0.50, 1.24)	
Black	ref	1.11 (0.62, 1.98)	0.73 (0.40, 1.34)	0.53 (0.29, 0.97)	
Mexican	ref	1.46 (0.73, 2.92)	0.91 (0.43, 1.93)	1.12 (0.52, 2.43)	
Hispanic	ref	1.01 (0.41, 2.46)	0.38 (0.12, 1.17)	0.56 (0.19, 1.64)	
Other race	ref	1.28 (0.59, 2.75)	1.17 (0.54, 2.55)	1.39 (0.66, 2.93)	
**Marital status**					0.08
Married/Living with partner	ref	1.03 (0.74, 1.45)	0.78 (0.55, 1.11)	0.94 (0.67, 1.34)	
Living alone	ref	0.73 (0.45, 1.20)	0.58 (0.34, 0.97)	0.56 (0.33, 0.93)	
**Education**					***0.04***
Less than 11th grade	ref	1.29 (0.62, 2.72)	1.05 (0.45, 2.45)	1.15 (0.52, 2.53)	
High school or GED	ref	1.01 (0.55, 1.85)	0.73 (0.39, 1.38)	0.49 (0.23, 1.03)	
Some college or AA degree above	ref	0.86 (0.61, 1.22)	0.66 (0.46, 0.94)	0.81 (0.58, 1.13)	
**Smoking status**					0.21
No	ref	1.05 (0.74, 1.50)	0.83 (0.58, 1.18)	0.83 (0.58, 1.19)	
Yes	ref	0.82 (0.52, 1.29)	0.60 (0.36, 0.99)	0.81 (0.51, 1.31)	
**Alcohol user**					0.13
Never	ref	0.70 (0.31, 1.58)	0.62 (0.25, 1.51)	0.91 (0.42, 1.95)	
Former	ref	1.27 (0.41, 3.94)	0.88 (0.22, 3.46)	1.18 (0.34, 4.07)	
Mild	ref	0.93 (0.63, 1.35)	0.75 (0.51, 1.10)	0.76 (0.52, 1.12)	
Heavy	ref	1.01 (0.61, 1.69)	0.64 (0.37, 1.12)	0.74 (0.42, 1.28)	
**Poverty to income ratio**					***0.04***
< 1.5	ref	1.22 (0.78, 1.91)	0.80 (0.48, 1.33)	0.89 (0.54, 1.47)	
1.5–3.5	ref	0.81 (0.51, 1.28)	0.53 (0.32, 0.88)	0.58 (0.36, 0.94)	
≥ 3.5	ref	0.82 (0.47, 1.40)	0.79 (0.47, 1.32)	0.87 (0.53, 1.46)	
**Pelvic Infection Disease**					0.23
No	ref	0.97 (0.73, 1.29)	0.71 (0.52, 0.96)	0.78 (0.58, 1.05)	
Yes	ref	0.63 (0.22, 1.80)	0.94 (0.32, 2.74)	0.70 (0.27, 1.81)	
**Age of menarche, years**					***0.04***
< 15	ref	0.96 (0.70, 1.30)	0.70 (0.50, 0.97)	0.88 (0.64, 1.20)	
≥ 15	ref	0.88 (0.47, 1.64)	0.82 (0.43, 1.56)	0.46 (0.23, 0.94)	

The model was adjusted for age(categorical), BMI (categorical), race (Mexican, Hispanic, White, Black, and Other race), marital status (living alone, and married/living with partner), education (Some college or AA degree above, High school or GED, and Less than 11th grade), smoking status (No, Yes), alcohol user (never, former, mild, and heavy) and PIR (< 1.5, 1.5–3.5, and ≥ 3.5), PID (No, Yes), and age of menarche (< 15 years or ≥ 15 years). All covariates in the subgroup analysis models were adjusted, excepting the stratification variable itself (for example, “age” was not included as a covariate in the age subgroup). *P* value in bold indicates statistical significance

*Abbreviation*: *BMI* body mass index, *OR* odds ratios, *CI* confidence interval, *ref* reference group, *PLR* platelet-lymphocyte ratio, *Q1* the first quartile, *Q2* the second quartile, *Q3* the third quartile, *Q4* the highest quartile

## Discussion

The findings showed that SII and PLR were negatively associated with infertility after adjusting for potential covariates. The increasing trend in SII, PLR were associated with a lower risk of infertility. No significant association was observed between LC, PPN, NLR, LMR and infertility.

It has been widely accepted that inflammation and immunity play critical role in many causes of female infertility, such as PCOS, endometriosis, genital and pelvic inflammatory diseases, as well as unexplained infertility. Moreover, immunological balance between tolerance of fetus and defense against infections is essential for successful pregnancy. Altered numbers or disturbed function of immune cells contribute to pathogenesis of reproductive adverse events, such as implantation failure, recurrent pregnancy loss and preterm birth [27]. Current diagnostic tests for causes of infertility are expensive, complicated and may cause discomfort to patients. However, several emerging parameters derived from peripheral blood, such as SII, LC, PPN, PLR, NLR and LMR have been accepted as inflammatory markers indicating inflammation and immune status and as possible predictors for immune related diseases with its cost-effective, rapid and convenient characteristics. Nevertheless, these indexes have become novel markers related to diverse pregnancy complications or neonatal outcomes, such as preeclampsia, gestational diabetes mellitus, spontaneous preterm birth, low placental and birth weights [28–31]. But limited direct evidence of link between these novel markers and infertility could be found.

One of our findings, consistent with earlier study demonstrating that imbalanced lymphocyte ratios or interaction between immune cells involve in the pathogenesis of infertility and disturbed pregnancy [9], was that LC level was elevated in the infertility group indicting LC might be associated with infertility. Higher level of CD19^+^ B lymphocytes was found in women with infertility and positive antiphospholipid antibodies when compared to healthy women or compared to women with a history of infertility without antiphospholipid antibodies [32]. Dysfunction of B lymphocytes with the production of antibodies among these women suggest that autoimmune process should be related to adverse obstetric outcomes.

For a more comprehensive assessment, we simultaneously assessed the associations between other inflammatory markers derived from PC, NC, MC, LC, and infertility. Interestingly, PPN, an emerging marker that has not been reported in literature about association between inflammatory markers and infertility, was positively related to risk of infertility in model adjusted for age in our study. It has been reported that PPN was associated positively with female estradiol [10]. Tang et al. found that PPN was negatively associated with BMD, indicating PPN may be a convenient marker that could be applied to predict the risk of osteoporosis for postmenopausal women [11]. Different findings of these two studies may be due to women included with different age. Thus, more investigations are needed to determine the association between PPN and reproductive function among women of reproductive age. Recently, a cross-sectional study revealed a significant positive correlation between NLR and PLR among infertility patients [33]. Jing X et al. reported that NLR was independent risk factors of endometriosis related infertility [34] based on a retrospective study comprising 662 women with endometriosis. Our study showed negative association between infertility and NLR, PLR based on a much larger sample size, but the relation between infertility and NLR had no statistical difference. Specific causes of infertility were not available in our study because they were not collected in NHANES datasets. Thus, we speculate that the difference between the two research findings may stem from different causes of infertility among the included population. Moreover, earlier studies have shown that LMR correlate with poor prognosis of patients with acute ischemic stroke and advanced soft tissue sarcoma [13, 14]. Previous cross-sectional study among Chinese women with normal pregnancy indicated that LMR level showed a gradual downward trend along with the trimesters of pregnancy [17]. We found positive association trend between LMR level and infertility in this study but without statistical significance for the first time. Thus, more researches are necessary to clarify the potential connection between LMR level and infertility.

Normal pregnancy is associated with a continuous systemic inflammatory response. The importance of SII has been emphasized because elevated SII has been considered as an effective blood indicator reflecting systemic inflammation conditions, such as increased risk of malignancy, depression, cognitive impairment, cardiopulmonary, rheumatic, and metabolic diseases [18, 35–42]. Nonetheless, the relationship between SII and infertility has not been reported yet. Findings on relationship between SII and reproductive health seemed to be controversial. Previous studies showed that clinical value of higher SII level for the prediction of miscarriage and adverse neonatal outcomes [43, 44], while SII showed dynamic changes during pregnant trimesters and the upper limit of SII in the second trimester showed the highest value [17]. The latter study supported the presumption that lower SII level may exert adverse influence on pregnancy, which is consistent with the negative relationship between SII and infertility in our study.

To our knowledge, this is the first study to assess the association between inflammatory markers including SII, LC, PPN, PLR, NLR, LMR and infertility based on the US NHANES data of women aged 20–45 years. We believe that this study is an important preliminary exploration of the relationship between inflammatory markers and infertility and has significant value for further research on the relationship and mechanism between inflammatory markers and infertility in the future. Findings from this study emphasized the clinical value of these markers, such as replacing current expensive standard tests for infertility diagnosis, predicting infertility prognosis and effects of assisted reproductive technology. Since these parameters are cheap, easy to apply and significantly associated with infertility prevalence, further study on the underlying mechanism of the relationship between these inflammatory markers and infertility should be performed.

There are several limitations in this study. First, the causality between risk of infertility and these inflammatory markers could not be established due to the cross-sectional design of this study. There may be intriguing interaction between infertility and inflammation immune response. Second, in the view of that some information was collected based on participants self-reported questionnaires, there might be recall bias in this study. Third, it is clear that male factors should be considered in infertility related research. However, semen analysis of the male partner and the specific causes of infertility were unavailable in this study, due to male related reproductive questionnaire was not collected in the NHANES database and female reproductive questionnaire also did not collect causes of infertility. In addition, prevalence of self-reported infertility in our study was 13.88%, which was similar to previous one [45], indicating sample in our study was representative for the US population. Finally, anti-Müllerian hormone (AMH) and basal sexual hormones have been regarded as essential measurements in the diagnosis and treatment of infertility. We failed to include AMH and basal sexual hormones as covariates in this study because they were unavailable in NHANES2013-2020 datasets. However, we did another analysis via collecting serum estradiol, total testosterone, and sex hormone binding globulin levels available in 2013–2016 NHANES datasets as parts of covariates and found the association between inflammatory markers and infertility remained consistent (results not shown).

## Conclusion

Taken together, findings in this study showed that SII and PLR were negatively associated with infertility and infertile women should be aware of SII, PLR. Further studies are needed to explore their association better and the underlying mechanisms.

### Supplementary Information

**Additional file 1: Supplementary Figure 1.** Distribution of inflammatory markers among individuals included. Legend: (A) SII; (B) LC; (C) PPN; (D) PLR; (E) NLR; (F) LMR were measured in 1×103 cells/μL. SII, systemic immune inflammation index; LC, lymphocyte count; PPN, product of platelet and neutrophil count; PLR, platelet to lymphocyte ratio; NLR, neutrophil to lymphocyte ratio; LMR, lymphocyte to monocyte ratio.

**Additional file 2: Supplementary Figure 2.** Distribution of log2-transformed inflammatory markers among individuals included. Legend: (A) log2-transformed SII; (B) log2-transformed LC; (C) log2-transformed PPN; (D)log2-transformed PLR; (E) log2-transformed NLR; (F) log2-transformed LMR. SII, systemic immune inflammation index; LC, lymphocyte count; PPN, product of platelet and neutrophil count; PLR, platelet to lymphocyte ratio; NLR, neutrophil to lymphocyte ratio; LMR, lymphocyte to monocyte ratio.

**Additional file 3: Supplementary Table 1.** Subgroup analyses for the relationship between log2-LC and infertility. Legend: Note: The model was adjusted for age(categorical), BMI (categorical), race (Mexican, Hispanic, White, Black, and Other race), marital status (living alone, and married/living with partner), education (Some college or AA degree above, High school or GED, and Less than 11th grade), smoking status (No, Yes), alcohol user (never, former, mild, and heavy) and PIR (<1.5, 1.5-3.5, and ≥3.5), PID (No, Yes), and age of menarche (<15 years or ≥15 years). All covariates in the subgroup analysis models were adjusted, excepting the stratification variable itself (for example, “age” was not included as a covariate in the age subgroup). *P* value in bold indicates statistical significance. Abbreviation: BMI, body mass index; OR, odds ratios; CI, confidence interval; ref, reference group. LC, lymphocyte count; Q1, the first quartile; Q2, the second quartile; Q3, the third quartile; Q4, the highest quartile. **Supplementary Table 2.** Subgroup analyses for the relationship between log2-PPN and infertility. Legend: Note: The model was adjusted for age(categorical), BMI (categorical), race (Mexican, Hispanic, White, Black, and Other race), marital status (living alone, and married/living with partner), education (Some college or AA degree above, High school or GED, and Less than 11th grade), smoking status (No, Yes), alcohol user (never, former, mild, and heavy) and PIR (<1.5, 1.5-3.5, and ≥3.5), PID (No, Yes), and age of menarche (<15 years or ≥15 years). All covariates in the subgroup analysis models were adjusted, excepting the stratification variable itself (for example, “age” was not included as a covariate in the age subgroup). *P* value in bold indicates statistical significance. Abbreviation: BMI, body mass index; OR, odds ratios; CI, confidence interval; ref, reference group; PPN, product of platelet and neutrophil count; Q1, the first quartile; Q2, the second quartile; Q3, the third quartile; Q4, the highest quartile. **Supplementary Table 3.** Subgroup analyses for the relationship between log2-NLR and infertility. Legend: Note: The model was adjusted for age(categorical), BMI (categorical), race (Mexican, Hispanic, White, Black, and Other race), marital status (living alone, and married/living with partner), education (Some college or AA degree above, High school or GED, and Less than 11th grade), smoking status (No, Yes), alcohol user (never, former, mild, and heavy) and PIR (<1.5, 1.5-3.5, and ≥3.5), PID (No, Yes), and age of menarche (<15 years or ≥15 years). All covariates in the subgroup analysis models were adjusted, excepting the stratification variable itself (for example, “age” was not included as a covariate in the age subgroup). *P* value in bold indicates statistical significance. Abbreviation: BMI, body mass index; OR, odds ratios; CI, confidence interval; ref, reference group; NLR, neutrophil-lymphocyte ratio; Q1, the first quartile; Q2, the second quartile; Q3, the third quartile; Q4, the highest quartile. **Supplementary Table 4.** Subgroup analyses for the relationship between log2-LMR and infertility. Legend: Note: The model was adjusted for age(categorical), BMI (categorical), race (Mexican, Hispanic, White, Black, and Other race), marital status (living alone, and married/living with partner), education (Some college or AA degree above, High school or GED, and Less than 11th grade), smoking status (No, Yes), alcohol user (never, former, mild, and heavy) and PIR (<1.5, 1.5-3.5, and ≥3.5), PID (No, Yes), and age of menarche (<15 years or ≥15 years). All covariates in the subgroup analysis models were adjusted, excepting the stratification variable itself (for example, “age” was not included as a covariate in the age subgroup). *P* value in bold indicates statistical significance. Abbreviation: BMI, body mass index; OR, odds ratios; CI, confidence interval; ref, reference group; LMR, lymphocyte-monocyte ratio; Q1, the first quartile; Q2, the second quartile; Q3, the third quartile; Q4, the highest quartile.

## Data Availability

The datasets supporting the conclusions of this article are available in the NHANES [https://www.cdc.gov/nchs/nhanes/].
