# Supplementary material for: Inflammatory markers are associated with infertility prevalence: a cross-sectional analysis of the NHANES 2013–2020
Source: BMC Public Health. 2024 Jan 18;24:221. doi: 10.1186/s12889-024-17699-4 (PMC10797998; doi:10.1186/s12889-024-17699-4)
Supplement: Supplementary file 1 — Additional file 1: Supplementary Figure 1. Distribution of inflammatory markers among individuals included. Legend: (A) SII; (B) LC; (C) PPN; (D) PLR; (E) NLR; (F) LMR were measured in 1×10^3^ cells/μL. SII, systemic immune inflammation index; LC, lymphocyte count; PPN, product of platelet and neutrophil count; PLR, platelet to lymphocyte ratio; NLR, neutrophil to lymphocyte ratio; LMR, lymphocyte to monocyte ratio. [file 12889_2024_17699_MOESM1_ESM.pdf]

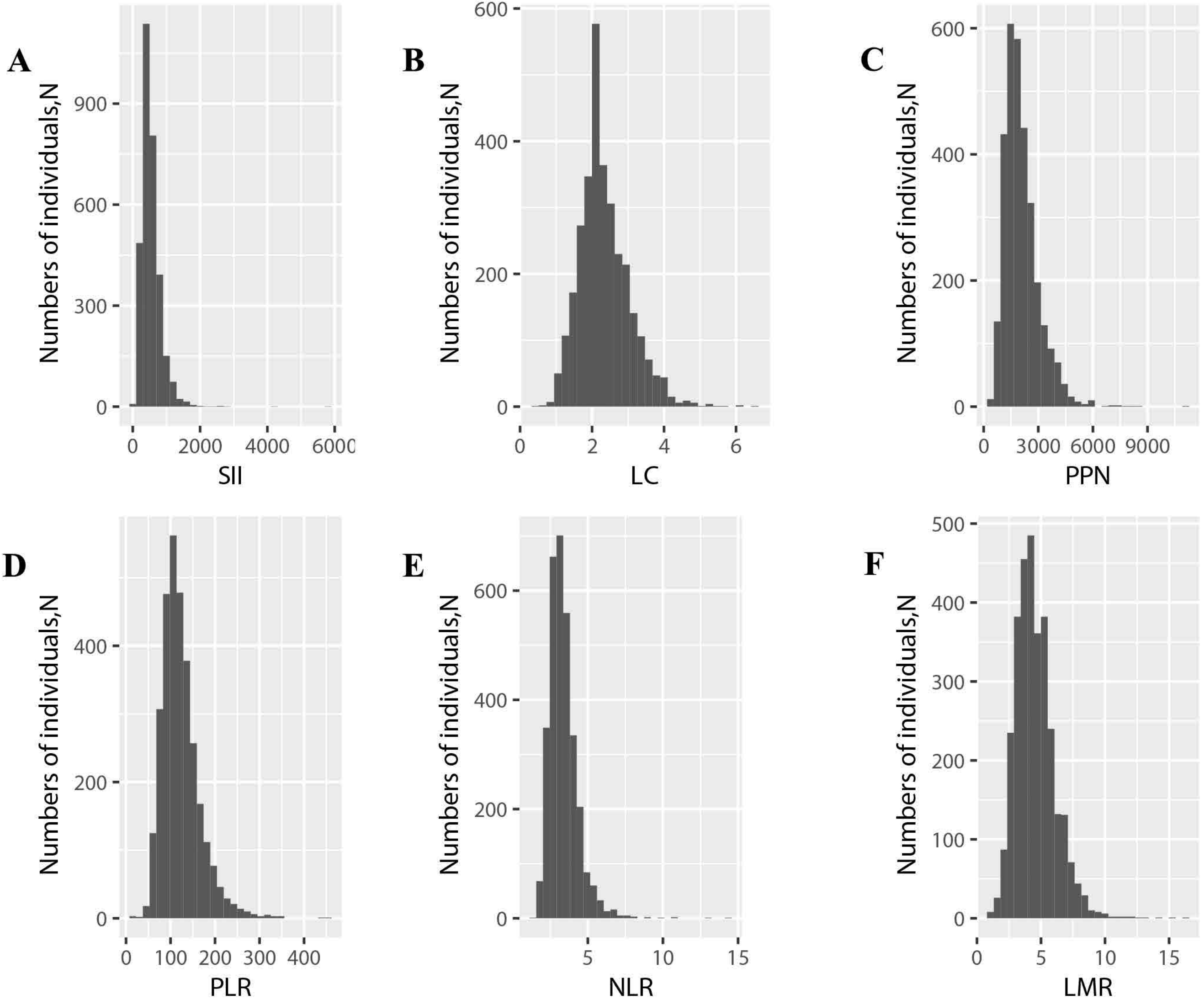
A
B
C
D
E
F
Numbers of individuals,N
SII
LC
PPN
PLR
NLR
LMR

Supplementary Figure 1 Distribution of inflammatory markers among individuals included.

(A)SII; (B) LC; (C) PPN; (D)PLR; (E) NLR; (F) LMR were measured in 1×1000 cells/μL. SII, systemic immune inflammation index; LC, lymphocyte count; PPN, product of platelet and neutrophil count; PLR, platelet to lymphocyte ratio; NLR, neutrophil to lymphocyte ratio; LMR, lymphocyte to monocyte ratio.
