# Supplementary material for: Inflammatory markers are associated with infertility prevalence: a cross-sectional analysis of the NHANES 2013–2020
Source: BMC Public Health. 2024 Jan 18;24:221. doi: 10.1186/s12889-024-17699-4 (PMC10797998; doi:10.1186/s12889-024-17699-4)
Supplement: Supplementary file 2 — Additional file 2: Supplementary Figure 2. Distribution of log2-transformed inflammatory markers among individuals included. Legend: (A) log2-transformed SII; (B) log2-transformed LC; (C) log2-transformed PPN; (D)log2-transformed PLR; (E) log2-transformed NLR; (F) log2-transformed LMR. SII, systemic immune inflammation index; LC, lymphocyte count; PPN, product of platelet and neutrophil count; PLR, platelet to lymphocyte ratio; NLR, neutrophil to lymphocyte ratio; LMR, lymphocyte to monocyte ratio. [file 12889_2024_17699_MOESM2_ESM.pdf]

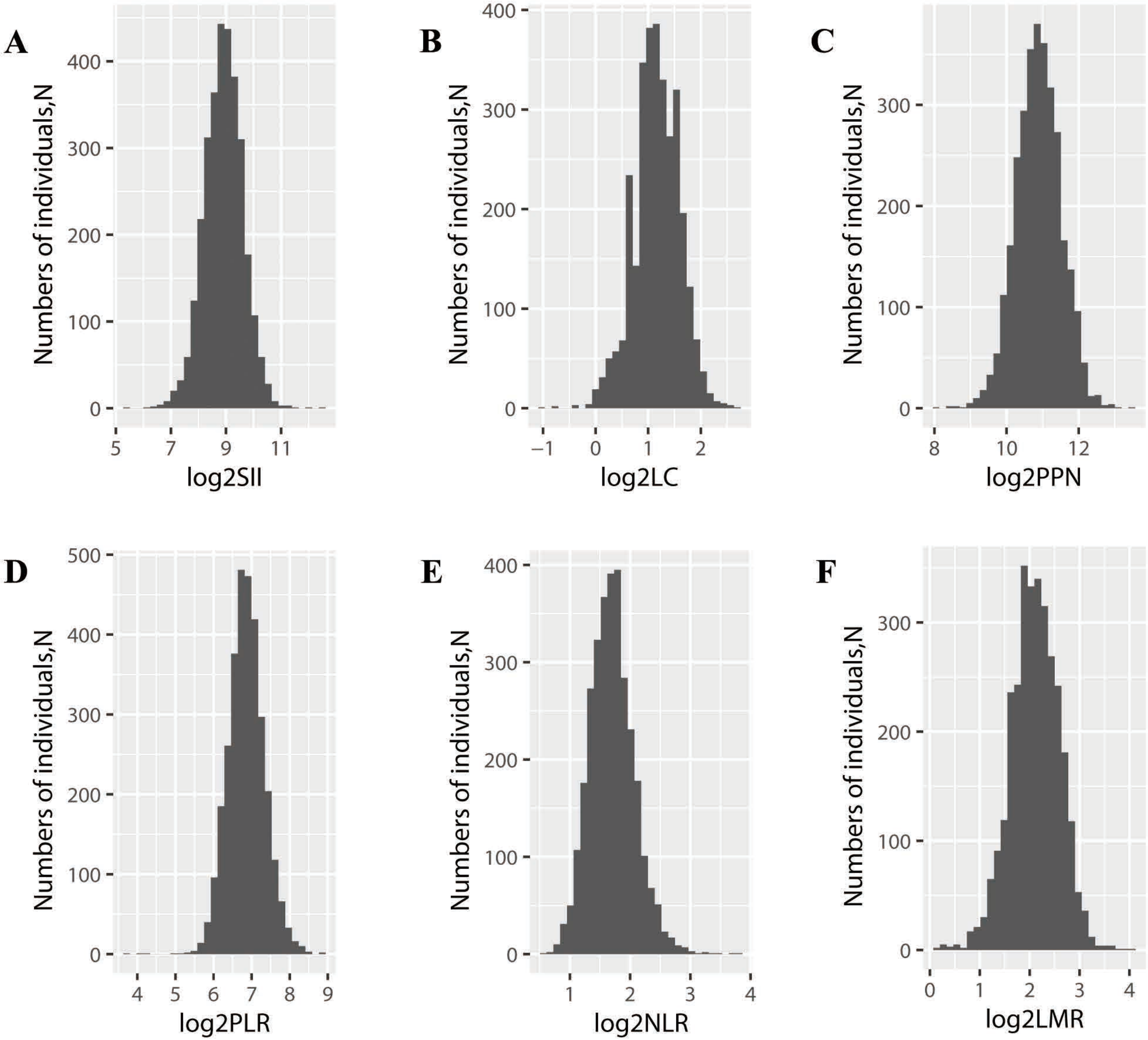
A
B
C
D
E
F
Numbers of individuals,N
log2SII
log2LC
log2PPN
log2PLR
log2NLR
log2LMR

Supplementary Figure 2 Distribution of log2-transformed inflammatory markers among individuals included.

(A) log2-transformed SII; (B) log2-transformed LC; (C) log2-transformed PPN; (D)log2-transformed PLR; (E) log2-transformed NLR; (F) log2-transformed LMR. SII, systemic immune inflammation index; LC, lymphocyte count; PPN, product of platelet and neutrophil count; PLR, platelet to lymphocyte ratio; NLR, neutrophil to lymphocyte ratio; LMR, lymphocyte to monocyte ratio.
