# Supplementary material for: Inflammatory markers are associated with infertility prevalence: a cross-sectional analysis of the NHANES 2013–2020
Source: BMC Public Health. 2024 Jan 18;24:221. doi: 10.1186/s12889-024-17699-4 (PMC10797998; doi:10.1186/s12889-024-17699-4)
Supplement: Supplementary file 3 — Additional file 3: Supplementary Table 1. Subgroup analyses for the relationship between log2-LC and infertility. Legend: Note: The model was adjusted for age(categorical), BMI (categorical), race (Mexican, Hispanic, White, Black, and Other race), marital status (living alone, and married/living with partner), education (Some college or AA degree above, High school or GED, and Less than 11th grade), smoking status (No, Yes), alcohol user (never, former, mild, and heavy) and PIR (<1.5, 1.5-3.5, and ≥3.5), PID (No, Yes), and age of menarche (<15 years or ≥15 years). All covariates in the subgroup analysis models were adjusted, excepting the stratification variable itself (for example, “age” was not included as a covariate in the age subgroup). P value in bold indicates statistical significance. Abbreviation: BMI, body mass index; OR, odds ratios; CI, confidence interval; ref, reference group. LC, lymphocyte count; Q1, the first quartile; Q2, the second quartile; Q3, the third quartile; Q4, the highest quartile. Supplementary Table 2. Subgroup analyses for the relationship between log2-PPN and infertility. Legend: Note: The model was adjusted for age(categorical), BMI (categorical), race (Mexican, Hispanic, White, Black, and Other race), marital status (living alone, and married/living with partner), education (Some college or AA degree above, High school or GED, and Less than 11th grade), smoking status (No, Yes), alcohol user (never, former, mild, and heavy) and PIR (<1.5, 1.5-3.5, and ≥3.5), PID (No, Yes), and age of menarche (<15 years or ≥15 years). All covariates in the subgroup analysis models were adjusted, excepting the stratification variable itself (for example, “age” was not included as a covariate in the age subgroup). P value in bold indicates statistical significance. Abbreviation: BMI, body mass index; OR, odds ratios; CI, confidence interval; ref, reference group; PPN, product of platelet and neutrophil count; Q1, the first quartile; Q2, the second qua [file 12889_2024_17699_MOESM3_ESM.docx]

**Supplementary Table 1 Subgroup analyses for the relationship between log2-LC and infertility.**

| **Subgroups** | **Q1 [-1.00, 0.93]** | **Q2 (0.93, 1.20]** | **Q3 (1.20, 1.43]** | **Q4 (1.43, 2.72]** | ***P* for interaction** |
| --- | --- | --- | --- | --- | --- |
| **Age, years** |  |  |  |  | ***0.03*** |
| <35 | ref | 0.83 (0.51, 1.33) | 1.37 (0.87, 2.14) | 1.80 (1.21, 2.68) |  |
| ≥35 | ref | 0.90 (0.61, 1.31) | 1.19 (0.81, 1.76) | 1.10 (0.76, 1.60) |  |
| **BMI, kg/m^2^** |  |  |  |  | ***0.05*** |
| <25 | ref | 0.90 (0.55, 1.46) | 0.89 (0.50, 1.60) | 1.06 (0.61, 1.84) |  |
| 25-30 | ref | 0.56 (0.28, 1.12) | 1.20 (0.65, 2.21) | 0.89 (0.47, 1.67) |  |
| ≥30 | ref | 0.88 (0.57, 1.38) | 1.25 (0.81, 1.92) | 1.36 (0.92, 2.02) |  |
| **Race** |  |  |  |  | ***< 0.01*** |
| White | ref | 0.69 (0.41, 1.13) | 1.03 (0.64, 1.68) | 1.60 (1.04, 2.46) |  |
| Black | ref | 2.42 (1.24, 4.70) | 3.50 (1.78, 6.86) | 2.24 (1.15, 4.35) |  |
| Mexican | ref | 0.51 (0.24, 1.11) | 0.62 (0.28, 1.39) | 1.08 (0.58, 2.02) |  |
| Hispanic | ref | 0.42 (0.14, 1.26) | 0.72 (0.27, 1.93) | 0.95 (0.38, 2.33) |  |
| Other | ref | 0.84 (0.43, 1.62) | 1.28 (0.66, 2.49) | 0.75 (0.37, 1.53) |  |
| **Marital status** |  |  |  |  | ***< 0.01*** |
| Married/Living with partner | ref | 0.60 (0.42, 0.86) | 1.00 (0.71, 1.42) | 1.09 (0.79, 1.51) |  |
| Living alone | ref | 1.96 (1.12, 3.44) | 2.14 (1.19, 3.85) | 2.45 (1.43, 4.19) |  |
| **Education** |  |  |  |  | ***0.03*** |
| Less than 11th grade | ref | 0.70 (0.32, 1.52) | 0.93 (0.42, 2.05) | 1.11 (0.53, 2.32) |  |
| High school or GED | ref | 0.91 (0.42, 1.98) | 2.17 (1.09, 4.32) | 1.87 (0.95, 3.65) |  |
| Some college or AA degree above | ref | 0.88 (0.62, 1.25) | 1.11 (0.77, 1.58) | 1.28 (0.93, 1.77) |  |
| **Smoking status** |  |  |  |  | ***0.02*** |
| No | ref | 0.84 (0.59, 1.19) | 1.24 (0.88, 1.75) | 1.06 (0.76, 1.49) |  |
| Yes | ref | 0.86 (0.50, 1.47) | 1.19 (0.69, 2.04) | 1.80 (1.14, 2.86) |  |
| **Alcohol user** |  |  |  |  | ***0.05*** |
| Never | ref | 0.74 (0.33, 1.70) | 0.81 (0.35, 1.85) | 0.83 (0.38, 1.82) |  |
| Former | ref | 0.62 (0.14, 2.85) | 1.50 (0.44, 5.10) | 1.41 (0.43, 4.67) |  |
| Mild | ref | 0.90 (0.61, 1.32) | 1.25 (0.84, 1.86) | 1.29 (0.90, 1.85) |  |
| Heavy | ref | 0.84 (0.47, 1.49) | 1.36 (0.78, 2.38) | 1.71 (1.02, 2.88) |  |
| **Poverty to income ratio** |  |  |  |  | ***<0.01*** |
| <1.5 | ref | 0.77 (0.46, 1.29) | 1.35 (0.84, 2.16) | 1.05 (0.65, 1.69) |  |
| 1.5-3.5 | ref | 0.96 (0.56, 1.63) | 1.11 (0.64, 1.93) | 2.06 (1.31, 3.22) |  |
| ≥3.5 | ref | 0.89 (0.54, 1.44) | 1.33 (0.80, 2.23) | 1.10 (0.67, 1.80) |  |
| **Pelvic Infection Disease** |  |  |  |  | 0.37 |
| No | ref | 0.84 (0.61, 1.14) | 1.25 (0.93, 1.70) | 1.34 (1.01, 1.77) |  |
| Yes | ref | 0.96 (0.35, 2.64) | 0.90 (0.31, 2.66) | 1.09 (0.42, 2.81) |  |
| **Age of menarche, years** |  |  |  |  | 0.13 |
| <15 | ref | 0.83 (0.60, 1.14) | 1.17 (0.85, 1.61) | 1.21 (0.89, 1.62) |  |
| ≥15 | ref | 0.96 (0.46, 1.97) | 1.52 (0.75, 3.08) | 2.01 (1.09, 3.72) |  |

Note: The model was adjusted for age(continuous), BMI (continuous), race (Mexican, Hispanic, White, Black, and Other race), marital status (living alone, and married/living with partner), education (Some college or AA degree above, High school or GED, and Less than 11th grade), smoking status (no, yes), alcohol user (never, former, mild, and heavy) and PIR (<1.5, 1.5-3.5, and ≥3.5), PID (no, yes), and age of menarche (<15 years or ≥15 years). All covariates in the subgroup analysis models were adjusted, excepting the stratification variable itself (for example, “age” was not included as a covariate in the age subgroup). *P* value in bold indicates statistical significance.

Abbreviation: BMI, body mass index; OR, odds ratios; CI, confidence interval; ref, reference group. LC, lymphocyte count; Q1, the first quartile; Q2, the second quartile; Q3, the third quartile; Q4, the highest quartile.

**Supplementary Table 2 Subgroup analyses for the relationship between log2-PPN and infertility.**

| **Subgroups** | **Q1 [8.00, 10.47]** | **Q2 (10.47, 10.89]** | **Q3 (10.89, 11.30]** | **Q4 (11.30, 13.42]** | ***P* for interaction** |
| --- | --- | --- | --- | --- | --- |
| **Age, years** |  |  |  |  | ***0.01*** |
| <35 | ref | 1.22 (0.78, 1.92) | 1.22 (0.77, 1.93) | 1.64 (1.06, 2.53) |  |
| ≥35 | ref | 1.51 (1.03, 2.22) | 0.98 (0.65, 1.46) | 0.98 (0.66, 1.47) |  |
| **BMI, kg/m2** |  |  |  |  | ***< 0.01*** |
| <25 | ref | 0.81 (0.50, 1.31) | 0.54 (0.29, 0.98) | 1.32 (0.76, 2.27) |  |
| 25-30 | ref | 1.51 (0.80, 2.87) | 1.04 (0.52, 2.07) | 1.26 (0.65, 2.47) |  |
| ≥30 | ref | 1.64 (1.01, 2.67) | 1.24 (0.77, 1.99) | 1.05 (0.65, 1.69) |  |
| **Race** |  |  |  |  | ***< 0.01*** |
| White | ref | 1.70 (1.03, 2.81) | 1.21 (0.71, 2.05) | 1.62 (0.98, 2.68) |  |
| Black | ref | 1.45 (0.84, 2.51) | 0.84 (0.44, 1.61) | 1.05 (0.57, 1.93) |  |
| Mexican | ref | 1.16 (0.49, 2.75) | 1.75 (0.79, 3.88) | 1.63 (0.74, 3.62) |  |
| Hispanic | ref | 1.06 (0.40, 2.83) | 0.59 (0.21, 1.66) | 0.58 (0.20, 1.69) |  |
| Other | ref | 0.92 (0.45, 1.86) | 1.03 (0.52, 2.03) | 1.04 (0.52, 2.07) |  |
| **Marital status** |  |  |  |  | 0.28 |
| Married/Living with partner | ref | 1.39 (0.98, 1.98) | 1.18 (0.82, 1.70) | 1.27 (0.88, 1.82) |  |
| Living alone | ref | 1.14 (0.68, 1.91) | 0.85 (0.49, 1.46) | 1.17 (0.70, 1.94) |  |
| **Education** |  |  |  |  | 0.06 |
| Less than 11th grade | ref | 1.85 (0.84, 4.08) | 1.00 (0.43, 2.33) | 1.74 (0.81, 3.74) |  |
| High school or GED | ref | 1.30 (0.64, 2.65) | 0.81 (0.37, 1.75) | 1.24 (0.62, 2.48) |  |
| Some college or AA degree above | ref | 1.25 (0.88, 1.77) | 1.15 (0.81, 1.64) | 1.13 (0.79, 1.63) |  |
| **Smoking status** |  |  |  |  | ***0.02*** |
| No | ref | 1.12 (0.79, 1.58) | 1.03 (0.72, 1.47) | 0.99 (0.69, 1.42) |  |
| Yes | ref | 1.98 (1.15, 3.41) | 1.19 (0.67, 2.10) | 1.83 (1.07, 3.11) |  |
| **Alcohol user** |  |  |  |  | ***<0.01*** |
| Never | ref | 1.22 (0.56, 2.64) | 1.07 (0.48, 2.37) | 0.62 (0.24, 1.59) |  |
| Former | ref | 3.47(0.92, 13.09) | 1.34 (0.35, 5.16) | 1.60 (0.43, 5.98) |  |
| Mild | ref | 1.07 (0.72, 1.58) | 1.05 (0.71, 1.57) | 1.20 (0.82, 1.76) |  |
| Heavy | ref | 1.85 (1.03, 3.35) | 1.11 (0.59, 2.07) | 1.59 (0.87, 2.89) |  |
| **Poverty to income ratio** |  |  |  |  | ***0.01*** |
| <1.5 | ref | 1.65 (1.01, 2.68) | 1.00 (0.59, 1.70) | 1.18 (0.71, 1.96) |  |
| 1.5-3.5 | ref | 1.14 (0.67, 1.94) | 1.49 (0.89, 2.49) | 1.37 (0.83, 2.28) |  |
| ≥3.5 | ref | 1.25 (0.76, 2.04) | 0.82 (0.49, 1.39) | 1.27 (0.76, 2.12) |  |
| **Pelvic Infection Disease** |  |  |  |  | 0.41 |
| No | ref | 1.31 (0.97, 1.78) | 1.07 (0.78, 1.46) | 1.20 (0.88, 1.63) |  |
| Yes | ref | 1.47 (0.50, 4.31) | 1.03 (0.34, 3.11) | 1.71 (0.59, 4.99) |  |
| **Age of menarche, years** |  |  |  |  | 0.11 |
| <15 | ref | 1.49 (1.08, 2.06) | 1.14 (0.81, 1.59) | 1.32 (0.96, 1.83) |  |
| ≥15 | ref | 0.82 (0.42, 1.58) | 0.85 (0.44, 1.67) | 0.94 (0.48, 1.82) |  |

Note: The model was adjusted for age(continuous), BMI (continuous), race (Mexican, Hispanic, White, Black, and Other race), marital status (living alone, and married/living with partner), education (Some college or AA degree above, High school or GED, and Less than 11th grade), smoking status (no, yes), alcohol user (never, former, mild, and heavy) and PIR (<1.5, 1.5-3.5, and ≥3.5), PID (no, yes), and age of menarche (<15 years or ≥15 years). All covariates in the subgroup analysis models were adjusted, excepting the stratification variable itself (for example, “age” was not included as a covariate in the age subgroup). *P* value in bold indicates statistical significance.

Abbreviation: BMI, body mass index; OR, odds ratios; CI, confidence interval; ref, reference group; PPN, product of platelet and neutrophil count; Q1, the first quartile; Q2, the second quartile; Q3, the third quartile; Q4, the highest quartile.

**Supplementary Table 3 Subgroup analyses for the relationship between log2-NLR and infertility.**

| **Subgroups** | **Q1 [0.61, 1.45]** | **Q2 (1.45, 1.69]** | **Q3 (1.69, 1.93]** | **Q4 (1.93, 3.86]** | ***P* for interaction** |
| --- | --- | --- | --- | --- | --- |
| **Age, years** |  |  |  |  | 0.06 |
| <35 | ref | 0.76 (0.50, 1.15) | 0.83 (0.55, 1.24) | 0.66 (0.43, 1.02) |  |
| ≥35 | ref | 0.97 (0.65, 1.45) | 0.92 (0.61, 1.38) | 1.12 (0.76, 1.66) |  |
| **BMI, kg/m2** |  |  |  |  | ***0.02*** |
| <25 | ref | 1.00 (0.60, 1.69) | 0.64 (0.36, 1.13) | 0.87 (0.51, 1.48) |  |
| 25-30 | ref | 0.91 (0.46, 1.80) | 1.32 (0.69, 2.52) | 1.31 (0.68, 2.56) |  |
| ≥30 | ref | 0.80 (0.53, 1.19) | 0.90 (0.61, 1.34) | 0.83 (0.56, 1.22) |  |
| **Race** |  |  |  |  | ***< 0.01*** |
| White | ref | 0.85 (0.51, 1.42) | 0.73 (0.44, 1.22) | 0.87 (0.53, 1.43) |  |
| Black | ref | 0.80 (0.47, 1.37) | 0.75 (0.40, 1.39) | 0.33 (0.15, 0.75) |  |
| Mexican | ref | 1.28 (0.58, 2.85) | 1.20 (0.53, 2.76) | 1.68 (0.78, 3.61) |  |
| Hispanic | ref | 0.74 (0.25, 2.15) | 1.02 (0.38, 2.76) | 0.89 (0.32, 2.43) |  |
| Other | ref | 1.01 (0.46, 2.20) | 1.43 (0.70, 2.96) | 1.51 (0.71, 3.19) |  |
| **Marital status** |  |  |  |  | ***< 0.01*** |
| Married/Living with partner | ref | 0.94 (0.66, 1.35) | 1.02 (0.72, 1.46) | 1.22 (0.86, 1.73) |  |
| Living alone | ref | 0.75 (0.46, 1.21) | 0.61 (0.37, 1.03) | 0.49 (0.29, 0.84) |  |
| **Education** |  |  |  |  | ***< 0.01*** |
| Less than 11th grade | ref | 1.85 (0.82, 4.18) | 1.58 (0.68, 3.67) | 1.08 (0.44, 2.62) |  |
| High school or GED | ref | 0.59 (0.30, 1.15) | 0.88 (0.46, 1.65) | 0.55 (0.29, 1.05) |  |
| Some college or AA degree above | ref | 0.86 (0.60, 1.22) | 0.83 (0.58, 1.18) | 1.08 (0.77, 1.52) |  |
| **Smoking status** |  |  |  |  | ***0.02*** |
| No | ref | 0.94 (0.66, 1.34) | 0.96 (0.67, 1.38) | 1.17 (0.83, 1.66) |  |
| Yes | ref | 0.77 (0.47, 1.26) | 0.75 (0.46, 1.22) | 0.57 (0.35, 0.93) |  |
| **Alcohol user** |  |  |  |  | ***< 0.01*** |
| Never | ref | 0.85 (0.35, 2.04) | 1.81 (0.83, 3.90) | 0.84 (0.33, 2.13) |  |
| Former | ref | 0.68 (0.20, 2.31) | 0.56 (0.15, 2.06) | 1.30 (0.43, 3.90) |  |
| Mild | ref | 0.99 (0.67, 1.45) | 0.94 (0.63, 1.39) | 1.16 (0.79, 1.69) |  |
| Heavy | ref | 0.74 (0.44, 1.27) | 0.61 (0.35, 1.05) | 0.55 (0.32, 0.95) |  |
| **Poverty to income ratio** |  |  |  |  | ***0.00*** |
| <1.5 | ref | 1.11 (0.69, 1.79) | 1.17 (0.72, 1.91) | 0.90 (0.55, 1.49) |  |
| 1.5-3.5 | ref | 0.80 (0.49, 1.30) | 0.76 (0.47, 1.22) | 0.66 (0.40, 1.08) |  |
| ≥3.5 | ref | 0.75 (0.44, 1.27) | 0.77 (0.45, 1.31) | 1.32 (0.81, 2.17) |  |
| **Pelvic Infection Disease** |  |  |  |  | 0.17 |
| No | ref | 0.86 (0.64, 1.16) | 0.85 (0.63, 1.15) | 0.91 (0.68, 1.22) |  |
| Yes | ref | 1.11 (0.39, 3.16) | 1.76 (0.62, 5.03) | 1.43 (0.50, 4.12) |  |
| **Age of menarche, years** |  |  |  |  | ***0.05*** |
| <15 | ref | 0.92 (0.67, 1.26) | 1.02 (0.75, 1.40) | 0.99 (0.72, 1.35) |  |
| ≥15 | ref | 0.75 (0.39, 1.43) | 0.48 (0.24, 0.97) | 0.74 (0.39, 1.40) |  |

Note: The model was adjusted for age(continuous), BMI (continuous), race (Mexican, Hispanic, White, Black, and Other race), marital status (living alone, and married/living with partner), education (Some college or AA degree above, High school or GED, and Less than 11th grade), smoking status (no, yes), alcohol user (never, former, mild, and heavy) and PIR (<1.5, 1.5-3.5, and ≥3.5), PID (no, yes), and age of menarche (<15 years or ≥15 years). All covariates in the subgroup analysis models were adjusted, excepting the stratification variable itself (for example, “age” was not included as a covariate in the age subgroup). *P* value in bold indicates statistical significance.

Abbreviation: BMI, body mass index; OR, odds ratios; CI, confidence interval; ref, reference group; NLR, neutrophil-lymphocyte ratio; Q1, the first quartile; Q2, the second quartile; Q3, the third quartile; Q4, the highest quartile.

**Supplementary Table 4 Subgroup analyses for the relationship between log2-LMR and infertility.**

| **Subgroups** | **Q1 [0.11, 1.81]** | **Q2 (1.81, 2.12]** | **Q3 (2.12, 2.43]** | **Q4 (2.43, 4.03]** | ***P* for interaction** |
| --- | --- | --- | --- | --- | --- |
| **Age, years** |  |  |  |  | ***0.03*** |
| <35 | ref | 1.23 (0.77, 1.96) | 1.52 (0.97, 2.38) | 1.48 (0.95, 2.32) |  |
| ≥35 | ref | 1.25 (0.86, 1.83) | 0.98 (0.66, 1.43) | 0.95 (0.63, 1.44) |  |
| **BMI, kg/m2** |  |  |  |  | ***< 0.01*** |
| <25 | ref | 1.32 (0.79, 2.19) | 1.05 (0.62, 1.79) | 0.78 (0.43, 1.40) |  |
| 25-30 | ref | 1.34 (0.72, 2.48) | 0.69 (0.35, 1.37) | 0.65 (0.32, 1.34) |  |
| ≥30 | ref | 1.08 (0.70, 1.68) | 1.44 (0.95, 2.18) | 1.47 (0.97, 2.22) |  |
| **Race** |  |  |  |  | ***< 0.01*** |
| White | ref | 1.60 (1.01, 2.55) | 1.52 (0.95, 2.44) | 1.36 (0.81, 2.29) |  |
| Black | ref | 1.45 (0.73, 2.88) | 1.63 (0.84, 3.15) | 1.83 (0.97, 3.46) |  |
| Mexican | ref | 1.56 (0.71, 3.39) | 1.16 (0.53, 2.56) | 1.10 (0.49, 2.50) |  |
| Hispanic | ref | 0.36 (0.11, 1.18) | 0.95 (0.38, 2.32) | 0.78 (0.30, 2.07) |  |
| Other | ref | 0.75 (0.38, 1.48) | 0.58 (0.30, 1.15) | 0.52 (0.26, 1.05) |  |
| **Marital status** |  |  |  |  | ***0.05*** |
| Married/Living with partner | ref | 1.28 (0.89, 1.82) | 1.09 (0.77, 1.54) | 0.93 (0.64, 1.34) |  |
| Living alone | ref | 1.11 (0.65, 1.88) | 1.15 (0.67, 1.97) | 1.47 (0.88, 2.47) |  |
| **Education** |  |  |  |  | ***0.02*** |
| Less than 11th grade | ref | 1.90 (0.86, 4.20) | 1.17 (0.51, 2.67) | 1.06 (0.44, 2.51) |  |
| High school or GED | ref | 0.72 (0.35, 1.47) | 1.16 (0.62, 2.17) | 1.21 (0.63, 2.31) |  |
| Some college or AA degree above | ref | 1.27 (0.89, 1.82) | 1.20 (0.84, 1.71) | 1.12 (0.78, 1.61) |  |
| **Smoking status** |  |  |  |  | ***< 0.01*** |
| No | ref | 1.00 (0.69, 1.43) | 1.17 (0.83, 1.66) | 0.83 (0.57, 1.20) |  |
| Yes | ref | 1.89 (1.15, 3.12) | 1.18 (0.70, 2.01) | 2.00 (1.22, 3.30) |  |
| **Alcohol user** |  |  |  |  | ***< 0.01*** |
| Never | ref | 0.84 (0.36, 1.96) | 1.19 (0.56, 2.55) | 0.50 (0.19, 1.30) |  |
| Former | ref | 1.22 (0.29, 5.14) | 0.81 (0.23, 2.90) | 1.71 (0.52, 5.56) |  |
| Mild | ref | 1.02 (0.69, 1.50) | 1.11 (0.76, 1.63) | 0.95 (0.64, 1.42) |  |
| Heavy | ref | 2.12 (1.20, 3.73) | 1.46 (0.80, 2.65) | 1.98 (1.09, 3.60) |  |
| **Poverty to income ratio** |  |  |  |  | ***0.01*** |
| <1.5 | ref | 1.13 (0.70, 1.85) | 1.02 (0.62, 1.67) | 1.06 (0.65, 1.74) |  |
| 1.5-3.5 | ref | 0.99 (0.59, 1.66) | 1.25 (0.77, 2.05) | 1.43 (0.87, 2.36) |  |
| ≥3.5 | ref | 1.64 (0.97, 2.78) | 1.25 (0.74, 2.11) | 0.90 (0.51, 1.59) |  |
| **Pelvic Infection Disease** |  |  |  |  | 0.09 |
| No | ref | 1.24 (0.91, 1.69) | 1.26 (0.93, 1.70) | 1.12 (0.82, 1.54) |  |
| Yes | ref | 1.09 (0.42, 2.81) | 0.60 (0.20, 1.81) | 1.40 (0.51, 3.86) |  |
| **Age of menarche, years** |  |  |  |  | ***< 0.01*** |
| <15 | ref | 1.30 (0.95, 1.79) | 1.16 (0.85, 1.60) | 0.92 (0.65, 1.30) |  |
| ≥15 | ref | 0.82 (0.39, 1.75) | 1.23 (0.61, 2.47) | 2.18 (1.16, 4.10) |  |

Note: The model was adjusted for age(continuous), BMI (continuous), race (Mexican, Hispanic, White, Black, and Other race), marital status (living alone, and married/living with partner), education (Some college or AA degree above, High school or GED, and Less than 11th grade), smoking status (no, yes), alcohol user (never, former, mild, and heavy) and PIR (<1.5, 1.5-3.5, and ≥3.5), PID (no, yes), and age of menarche (<15 years or ≥15 years). All covariates in the subgroup analysis models were adjusted, excepting the stratification variable itself (for example, “age” was not included as a covariate in the age subgroup). *P* value in bold indicates statistical significance.

Abbreviation: BMI, body mass index; OR, odds ratios; CI, confidence interval; ref, reference group; LMR, lymphocyte-monocyte ratio; Q1, the first quartile; Q2, the second quartile; Q3, the third quartile; Q4, the highest quartile.
